# The Expression Patterns of c-Fos and c-Jun Induced by Different Frequencies of Electroacupuncture in the Brain

**DOI:** 10.1155/2015/343682

**Published:** 2015-09-29

**Authors:** Zheng-Ying Qiu, Yi Ding, Lu-ying Cui, Man-Li Hu, Ming-Xing Ding

**Affiliations:** College of Veterinary Medicine, Huazhong Agricultural University, Wuhan 430070, China

## Abstract

To investigate patterns of c-Fos and c-Jun expression induced by different frequencies of electroacupuncture (EA) in the brain, goats were stimulated by EA of 0, 2, 60, or 100 Hz at a set of “Baihui, Santai, Ergen, and Sanyangluo” points for 30 min. The pain threshold was measured using the potassium iontophoresis method. The levels of c-Fos and c-Jun were determined with Streptavidin-Biotin Complex immunohistochemistry. The results showed that the pain threshold induced by 60 Hz was 82.2% higher (*P* < 0.01) than that by 0, 2, or 100 Hz (6.5%, 35.2%, or 40.9%). EA induced increased c-Fos and c-Jun expression in most analgesia-related nuclei and areas in the brain. Sixty Hz EA increased more c-Fos or c-Jun expression than 2 Hz or 100 Hz EA in all the measured nuclei and areas except for the nucleus accumbens, the area septalis lateralis, the caudate nucleus, the nucleus amygdala basalis, and the locus coeruleus, in which c-Fos or c-Jun expressions induced by 60 Hz EA did not differ from those by 2 Hz or 100 Hz EA. It was suggested that 60 Hz EA activated more extensive neural circuits in goats, which may contribute to optimal analgesic effects.

## 1. Introduction

Acupuncture is a traditional therapeutic technique in oriental medicine, with a long history of approximately 3000 years. In the 1970s, electroacupuncture (EA), a modern version of acupuncture, began to replace manual manipulation because EA has better analgesic effects and its stimulation can be objectively quantified and controlled [1]. EA has been widely used for treating various pathologic conditions. Among them, its analgesic effects have rapidly gained interest and have been confirmed by numerous clinical observations and experimental studies. Studies have showed that EA can induce analgesia through the activation of the opioidergic, norepinephrinergic, dopaminergic, serotonergic, or cholinergic pathways in the central nerve system (CNS) [2–4]. Han and Wang [5] found that 2 Hz EA activated the arcuate nucleus (ARC) and caused the release of *β*-endorphin while 100 Hz EA motivated the parabrachial nucleus (PBN) to release dynorphin. Obviously, analgesia induced by different frequencies of EA occurs in distinct neural pathways. Although several investigators have determined the roles of individual cerebral nuclei or areas in EA-induced analgesia (EAA) with classical physical approaches (the stimulation and abolition of certain nerve fibres or nuclei) as well as pharmacological approaches (assessing antagonists and antibodies) [6–8], the neuronal circuitry underlying analgesia induced by different frequencies of EA is not yet clear.

C-fos and c-jun are two important immediate early genes that are rapidly and transiently expressed in cells after specific stimulations [9, 10]. Their products, C-Fos and C-Jun, can form dimer complexes, which serve as transcription factors by binding to the activator protein-1 site of target genes in a variety of neurons [11]. C-Fos and c-Jun have been considered to be valuable markers for the identification of neuronal activation and thus have been used to map functionally related neuronal pathways in the CNS in response to peripheral sensory neuronal activation [9, 10]. In addition, the target genes of the c-Fos and c-Jun complex have been verified and included those of some active substances participating in EAA in the CNS, such as preproenkephalin [12, 13], preprodynorphin [14], proopiomelanocortin [15, 16], cholecystokinin [17], and 5-TH receptors [18, 19]. Therefore, the detection of c-Fos and c-Jun expression is an advanced identification method for EA-activated nuclei or areas in the CNS.

Studies have shown that EA, in combination with an anaesthetic, resulted in the reduction of the dosage of the anaesthetic in humans, rats, and goats by 40%–46%, 50%, and over 75%, respectively, with the same analgesic effect as that caused by the application of the anaesthetic alone [20–22]. It is clear that the analgesic effect induced by EA in goats (ruminants) is superior to that in rats or human, and thus ruminants are optimal animal models for studies of the mechanisms of EAA. In the present study, the activated analgesia-related nuclei and areas in the goats' brains stimulated by different frequencies of EA were determined on the basis of the expression levels of c-Fos and c-Jun to investigate the neural mechanisms of EAA.

## 2. Materials and Methods

### 2.1. Animal Preparation

The study was conducted under the guidelines approved by the Animal Care Center, Huazhong Agricultural University.

Thirty-five 2-year-old hybrid male goats, weighing 25 ± 2 kg, were randomly allocated into 5 treatment groups (the blank control, 0 Hz, 2 Hz, 60 Hz, and 100 Hz) with 7 goats per group. All goats were purchased from the Hubei Agricultural Academy of Science, were fed by a dry grass diet supplemented with a cereal-based concentrate, and drank freely. They were dewormed and accustomed to the surroundings for two weeks. The experiment was performed in a quiet environment after 12 h of fasting, with a temperature of 20–25°C.

### 2.2. Electroacupuncture Procedures

A set of “Baihui,” “Santai,” “Ergen,” and “Sanyangluo” points was selected, which have traditionally been used in veterinary medicine to achieve anaesthesia for various parts of the body during surgery. The anatomic location and use of these points has been described in detail in veterinary medicine [23]. The “Baihui” point was identified on the dorsal midline between the spinous processes of the last lumbar and the first sacral vertebrae (but Baihui point in humans is located at the top of the skull). The “Santai” point was identified on the dorsal midline between the spinous processes of the fourth and fifth thoracic vertebrae. The “Ergen” points were identified bilaterally, with each at the pit ventrocaudal to the ear base between the ear base and the cranial border of the transverse process of the atlas on each side. The “Sanyangluo” points were also identified bilaterally, with each at approximately 5 cm ventral to the lateral tuberosity of the radius in the groove between the common digital extensor and the lateral digital extensor muscles of the forelimb. The “Baihui” and “Santai” points on the dorsal midline and the “Ergen” and “Sanyangluo” points on the right side of the body were used in this study. The acupoint sites were shaved and disinfected. The acupuncture needle (0.45 mm in diameter and 5 cm in length) was inserted perpendicularly into the “Baihui” point at a depth of approximately 3 cm. For the “Santai” point, the needle was inserted at a 45° angle at a depth of approximately 4 cm. These two needles shared a pair of wires from one output of the WQ-6F Electronic Acupunctoscope (Beijing Xindonghua Electronic Instrument Co., Ltd., Beijing, China). One needle (0.45 mm in diameter and 7.5 cm in length) was inserted into the right “Ergen” point to reach the subcutaneous tissue of the right temporal fossa. Another needle was inserted at approximately a 30° angle in a ventromedial direction into the right “Sanyangluo” point to reach the subcutaneous tissue of the medial side of the right forelimb. The two needles shared a pair of wires from another output of the same machine, and the parallel output option was selected. The intensity (approximate 3.2 V) remained constant during the entire EA procedure. The experimental goats were stimulated with EA at 0 (sham control), 2, 60, or 100 Hz for 30 min. The goats in the blank control were restrained in the same manner as the EA-treated goats but without needling and electric stimulation.

### 2.3. Determination of the Pain Threshold

The pain threshold was measured on the centre of the right flank using potassium iontophoresis [20] by a direct current induction therapy apparatus (Shantou Medical Equipment Factory Co., Ltd., Shantou, China). The pain was induced by potassium iontophoresis through a gradual increase in potassium ions (an effective pain stimulus) passing through the skin; the ion amount into the subcutaneous tissues was positively proportional to the increase in voltage or current. One skilled person who was blinded to the goat assignments assessed the pain threshold. The site of the pain threshold was shaved and cleaned with soap and water and disinfected with 75% ethanol. Two electrodes were soaked with saturated potassium chloride and placed 1-2 cm apart on the skin. The direct current induction therapy apparatus was used to deliver the pulsed direct current to the electrodes, which forced potassium ions into the subcutaneous tissues. The voltage was increased stepwise. The voltage level was recorded at the moment when obvious contractions of the local skin and muscle along with the animal's head turning toward the abdomen, back hunching, and eluding movements were observed. Then, the current was turned off. The pain threshold measurements of the goats were taken every 5 min for 3 times immediately before the insertion of the needle or after EA. The mean voltages before and after EA were expressed as *V*_0_ and *V*_*n*_, respectively. The percentage change in the pain threshold was calculated as follows: Δ(%) = (*V*_*n*_ − *V*_0_)/*V*_0_ × 100%.

### 2.4. Immunohistochemistry

**Figure 1:**
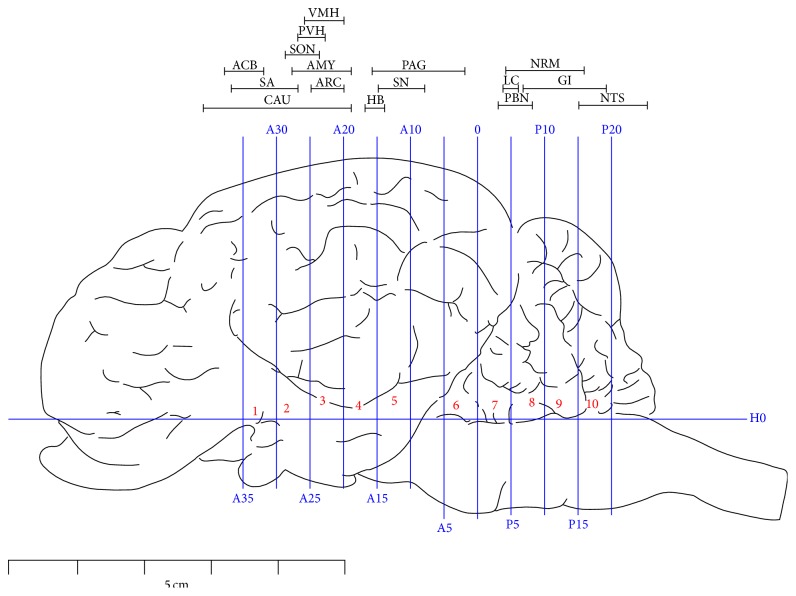
Brain sectioning. H0: the horizontal zero plane. A35, A25, A20, A15, A10, A5, and 0 show transverse planes at 35, 30, 25, 20, 15, 10, 5, and 0 mm rostral to the interaural line, and P5, P10, P15, and P20 show transverse planes at 5, 10, 15, and 20 mm caudal to the interaural line, respectively. The locations of nuclei or areas in the brain blocks are presented at the top of the figure. The nuclei and areas identified include the nucleus accumbens (ACB), the septal area (SA), the caudate nucleus (CAU), the supraoptic nucleus (SON), the paraventricular nucleus of the hypothalamus (PVH), the ventromedial nucleus of the hypothalamus (VMH), the arcuate nucleus (ARC), the amygdala (AMY), the habenular nucleus (HB), the periaqueductal grey (PAG), the substantia nigra (SN), the parabrachial nucleus (PBN), the locus coeruleus (LC), the nucleus raphe magnus (NRM), the gigantocellular reticular nucleus (GI), and the nucleus tractus solitarius (STN). The symbols in Figure 2 and Table 1 have the same meanings.

Once the pain threshold was measured, the goats were anaesthetized with intravenously administered xylidinothiazoline at 3 mg/kg. Physiological saline was infused through the bilateral carotid arteries and the blood flew out from the jugular vein. When the vein blood became transparent, 4% paraformaldehyde was infused to replace the saline and maintained for 1 h. Then, the heads of the goats were severed from the bodies and mounted in a stereotaxic instrument [24, 25]. In brief, the vertical interaural plane represented the zero reference point for the anterior-posterior coordinates, and the horizontal zero plane (H0) intersected the interaural point and a point 25 mm above the lower margin of the orbit. Six stainless steel marker tubes (0.8 mm in diameter) were inserted perpendicularly into the brain through predrilled holes along the midsagittal plane of the skull by means of stereotaxic electrode carriers at anterior 30 mm (A30), anterior 20 mm (A20), anterior 10 mm (A10), and 0 (0) and posterior 10 mm (P10) and posterior 20 mm (P20), respectively. The brains were immersed in 4% paraformaldehyde for 48 h. Then, the brain was removed from the skull, leaving the marker tubes in position. The brain and a part of the adjacent spinal cord were divided into several blocks according to the marker tubes as illustrated in Figure 1.

**Figure 2:**
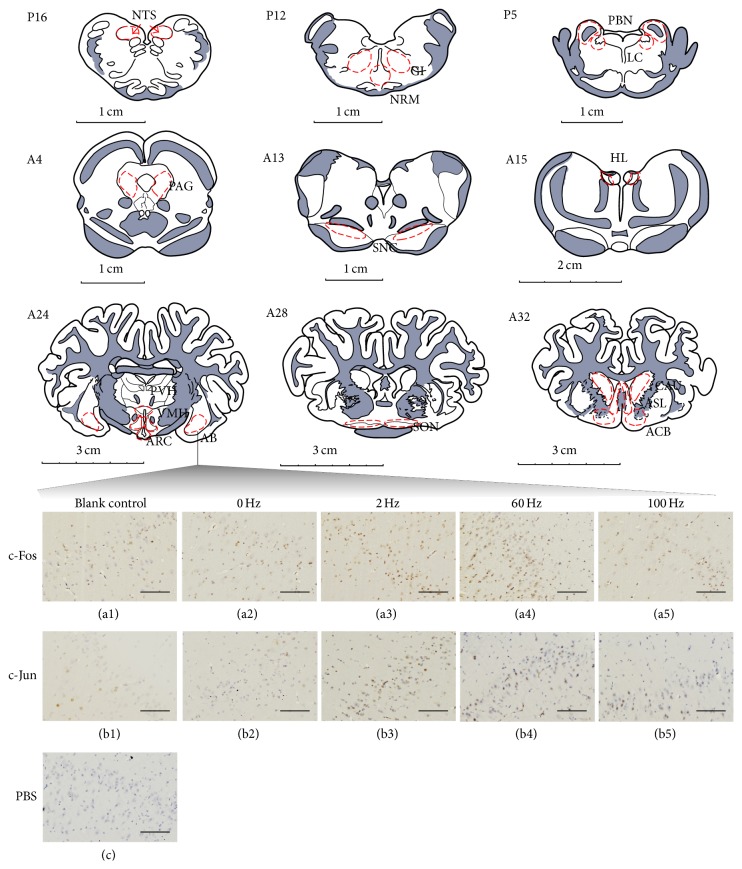
The nuclei (areas) locations used for the c-Fos-IR and c-Jun-IR counts and the representative c-Fos-IR and c-Jun-IR neurons in AB. P16: NTS at interaural levels of −16 mm. P12: NRM and GI at interaural levels of −12 mm. P5: PBN and LC at interaural levels of −5 mm. A4: the caudal ventrolateral periaqueductal grey (PAG) at interaural levels of 4 mm. A13: the pars compacta of the substantia nigra (SNC) at interaural levels of 13 mm. A15: the nucleus habenula lateralis (HL) at interaural levels of 16 mm. A24: PVH, VMH, ARC, and nucleus amygdala basalis (AB) at interaural levels of 24 mm. A28: SON at interaural levels of 28 mm. A32 shows ACB, the area septalis lateralis (ASL), and CAU at interaural levels of 32 mm. (a1)–(a5) show positive c-Fos-IR neurons in AB in the blank control and at 0, 2, 60, and 100 Hz, respectively. (b1)–(b5) show positive c-Jun-IR neurons in AB in the blank control and at 0, 2, 60, and 100 Hz, respectively. (c) shows the negative control. The bars = 100 *μ*m.

Part of the cortex and cerebellum were removed. Blocks 1–10 (B1–10) were embedded in paraffin with their rostral surface facing up. The locations of the nuclei or areas and the morphological characteristics of the neurons were identified according to the brain atlas of goats and pigs [24–27]. The nuclei or areas to be observed were the nucleus accumbens (ACB), the area septalis lateralis (ASL) and the caudate nucleus (CAU) in B1, the supraoptic nucleus (SON) in the caudal part of B2, the paraventricular nucleus of the hypothalamus (PVH), the ventromedial nucleus of the hypothalamus (VMH), ARC and the nucleus amygdala basalis (AB) in B3, the nucleus habenula lateralis (HL) in the caudal part of B4, pars compacta of the substantia nigra (SNC) in B5, the caudal ventrolateral periaqueductal grey (PAG) in B6, PBN and the locus coeruleus (LC) between the caudal part of B7 and the rostral part of B8, the nucleus raphe magnus (NRM) and the gigantocellular reticular nucleus (GI) in B9, and the nucleus tractus solitarius (NTS) in B10 (Figure 1). Each of the blocks was consecutively sectioned at a thickness of 5 *μ*m. Six serial sections of each nucleus or brain area were collected and mounted on poly-lysine coated slides. The slides were deparaffinized and rehydrated sequentially, followed by the Streptavidin-Biotin Complex immunohistochemistry procedure as follows. The serial slides were incubated with rabbit-anti-c-Fos (Wuhan Boster Biological Technology Ltd., Wuhan, China; 1:50 diluted in PBS), rabbit-anti-c-Jun (Santa Cruz Biotechnology, CA, USA; 1:50 diluted in PBS), and PBS (the negative control), respectively, and, sequentially, for 24 h at 4°C. Then, they were treated with secondary antibodies (SA1022-anti-rabbit IgG Kit, Wuhan Boster Biological Technology Ltd., Wuhan, China). Avidin-biotin complex Staining (Wuhan Boster Biological Technology Ltd., Wuhan, China) was visualized with diaminobenzidine for 7 min at room temperature. Finally, the sections were counterstained for nuclei with haematoxylin solution. The locations of the observed nuclei (areas) with the representative stained neurons (positive: yellow brown; negative: blue) are shown in Figure 2.

### 2.5. Cell Counting and Statistical Analysis

The slides were visualized with the images of the bilateral stained nuclei or areas under a light microscope (Nikon ECLIPSE 80I, Nikon Corporation, and Tokyo, Japan), and 3 fields of each nucleus or brain area on each side were observed with a 20 × objective lens. The numbers of c-Fos-like immunoreactive (c-Fos-IR) neurons or c-Jun-like immunoreactive (c-Jun-IR) neurons were counted with the Image-Pro plus 6.0 system (Media Cybernetics, Inc., Bethesda, MD, USA). The mean values calculated from each nucleus or brain area represented the c-Fos-IR or c-Jun-IR positive neurons per goat.

Statistical analysis was undertaken with SPSS 17.0 software (SPSS Inc., Chicago, USA). Data from the study were subjected to one-way ANOVA, followed by Bonferroni's post hoc test. Statistical significance was evaluated by determining whether *P* ≤ 0.05. All data are presented as the mean ± SD.

## 3. Results

### 3.1. Analgesic Effects Induced by Different Frequencies

**Figure 3:**
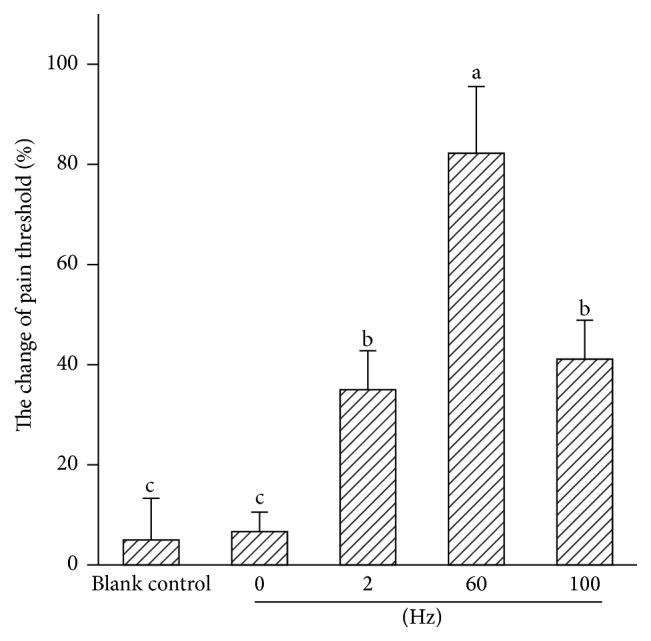
The changes in the pain threshold of goats stimulated by different frequencies (mean ± SD, %, *n* = 7). The values with different letters differ significantly (*P* < 0.01).

The analgesic effects of EA are represented by the change in the pain threshold (Figure 3). Compared with the blank control, the pain thresholds increased (*P* < 0.01) after the goats were treated with 2, 60, and 100 Hz for 30 min. The pain thresholds of goats receiving 100, 60, 2, and 0 Hz increased by 40.9%  ± 7.7% (9.00 ± 1.67 V to 12.62 ± 1.96 V), 82.2%  ± 13.4% (8.81 ± 1.67 V to 15.90 ± 2.22 V), 35.2%  ± 7.5% (8.38 ± 1.13 V to 11.29 ± 1.21 V), and 6.5%  ± 3.9% (7.67 ± 1.20 V to 8.14 ± 1.17 V), respectively. The pain thresholds of goats stimulated with 60 Hz were higher (*P* < 0.01) than that of goats receiving 100, 2, or 0 Hz.

### 3.2. The Expression Levels of c-Fos and c-Jun Induced by Different Frequencies in the Brain

C-Fos-IR and c-Jun-IR cells were observed in the measured nuclei and areas (Table 1). There was no difference (*P* > 0.05) in the numbers of c-Fos-IR or c-Jun-IR positive cells between the blank control and the 0 Hz treated goats. Two Hz EA induced c-Fos-IR cells to increase (*P* < 0.01) in ARC, PBN, NRM, HL, and GI and c-Jun-IR cells to increase (*P* < 0.01) in ACB and both to increase in AB, PVH, VMH, PAG, NTS, and LC. Sixty Hz EA induced the c-Fos-IR and c-Jun-IR cells to increase in the measured nuclei or areas with the exception of c-Jun-IR cells in HL. One hundred Hz EA induced c-Fos-IR cells to increase in HL, PAG, SNC, PBN, LC, NRM, NTS, and GI and c-Jun-IR cells to increase in ACB, ASL, CAU, SON, PVH, VMH, ARC, PAG, PBN, LC, GI, and NTS.

The levels of c-Fos or c-Jun expression induced by 60 Hz EA were higher than those induced by 2 Hz EA or 100 Hz EA in most measured nuclei and areas. Compared with 0 Hz EA, 60 Hz EA induced increases of c-Fos-IR cells by 6.62-, 4.11-, 4.07-, and 3.99-fold in HL, ARC, AB, and LC, respectively, and by 3.91- to 2.10-fold in the other measured nuclei or areas. Sixty Hz EA increased the C-Jun levels by 3.74-, 3.42-, 3.39-, and 2.75-fold in ARC, VMH, SON, and PBN, respectively, and by 2.68- to 1.25-fold in AB, ACB, CAU, PAG, NTS, ASL, PVH, LC, GI, NRM, SNC, and HL.

## 4. Discussion

A potent analgesic effect induced by EA depends on the proper prescription of specific acupoints. “Zusanli” (ST36) and “Sanyinjiao” (SP6), “Hegu” (LI4), and “Sanyangluo” (TE8) acupoints are commonly chosen for EA to relieve pain in rats, rabbits, and humans [28–30]. A few sets of acupoints have been employed for EAA in ruminants. Studies showed that EA at a set of “Baihui, Santai, Ergen, and Sanyangluo” acupoints (here using the Pinyin Naming System instead of the Meridian Numbering System because animal's meridians are not completely recorded) elicited an effective analgesia in cattle [31]. Liu et al. [20] demonstrated that EA at this set of acupoints caused a potent analgesic effect in goats. Frequency is another important factor responsible for EAA. Wang et al. [32] used EA to stimulate ST36 and “Kunlun” (BL60) in rabbits and found that better analgesia was induced by 2 Hz, followed by 30 Hz > 60 Hz > 100 Hz. Han et al. [6] and Chen et al. [33] reported that 100 Hz EA stimulation at ST36 and SP6 induced more effective analgesia than 2 Hz EA in rats. Cheng et al. [34] used different frequencies (2, 40, 60, 80, and 100 Hz) to stimulate a set of “Baihui, Santai, Ergen, and Sanyangluo” acupoints in goats and found that 60 Hz induced more potent analgesia compared to any other frequencies. These studies showed that analgesia induced by different frequencies seems to vary in species. In the present study, we used different frequencies (2, 60, and 100 Hz) to stimulate the set of “Baihui, Santai, Ergen, and Sanyangluo” acupoints in goats and found that the analgesia induced by 60 Hz was better than that by 2 or 100 Hz, which is consistent with the findings reported by Cheng et al. [34, 35].

Studies have shown that different frequencies of EA administered to the same acupuncture point activated different peptidergic, monoaminergic, or cholinergic systems in the CNS [2–4]. Some researchers investigated the effect of acupuncture stimulation on cerebral activation in humans with functional magnetic resonance imaging (fMRI) and have demonstrated that different frequencies activated different networks of cortical and brainstem structures and might mediate analgesia through modulating these corresponding neural networks [36, 37]. Studies with retrograde and anterograde tracer labelling showed that specific links exit among the neurons of these nuclei or areas [38, 39]. However, the analgesia-related neural circuitries activated by EA have not yet been clarified.

The c-Fos and c-Jun in different animal species have been proven to be reliable markers for identifying the nuclei activated by a variety of peripheral stimulations [9, 40]. In laboratory animals, acupuncture must be performed on either anaesthetized or, if unanesthetized, restrained subjects. Both treatments can upregulate c-Fos expression in several CNS areas [41, 42], representing a major confounding variable for the assessment of EA-induced c-Fos expression. However, immobilization-induced c-Fos expression can be markedly reduced after repeated restraint manoeuvres [43, 44]. The patterns of neuronal activity induced by habituation to repeated restraints are specific, allowing the effect of EA to be observed in rats previously submitted to repeated restraints [45]. In our study, the goats were allowed to habituate to the restraint and the environment for 2 weeks, thus reducing the interference of the restraint with the evaluation of the specific effects of acupuncture in unanesthetized animals. Several studies have shown that some nuclei of the hypothalamus, such as the ventromedial and paraventricular nuclei, are activated by stimuli linked to gastric filling or fasting [46]. Ruminants usually ingest food to filling for approximately 2 h, ruminate 0.5–1 h after feeding, and last for approximately 6–8 h. This ingestion includes the mechanical dissociation of food in the mouth after microbial predigestion in the rumen. To avoid individual variations in the stimuli from the upper digestive tract for the prehension and the mechanical dissociation of food, the goats were fasted for 12 h.

Both c-Fos and c-Jun can be rapidly expressed in the spinal cord dorsal horn (SCDH) neurons after both noxious and acupuncture stimulations. Studies have shown that nociceptive stimulation caused c-Fos expression to increase in laminae I and II of the SCDH at 20 min, significantly shifted to the laminae V and VI at 5-6 h, and returned to normal levels within 1 to 3 weeks [47]. In the pathological conditions, c-Fos or c-Jun expression may be important for the development of a pain state as part of the adaptive response of the spinal cord to continuous or subsequent nociceptive input or both. However, EA-induced c-Fos-IR neurons are restricted to laminae III and IV of the SCDH [48], which is somewhat different from that caused by the noxious stimulation in the SCDH. Both high and low frequencies were able to suppress c-Fos expression in the SCDH induced by the noxious stimulation [41]. Therefore, C-Fos or c-Jun levels are commonly used to evaluate how EA modifies neuronal activities caused by inflammatory or neuropathic pain in rats.

Supraspinal c-Fos or c-Jun expression is scarcely reported compared to that at the spinal level. Pan et al. [49, 50] found that noxious stimulation (immersing the footpad in 52°C water) and EA (4 Hz) both increased c-Fos expression in the anterior lobe of the pituitary gland and in the ARC as well as in nearby hypothalamic nuclei, such as PVH, VMH, medial preoptic area, and lateral hypothalamic area. These results suggest that there is probably a partial overlap of the central pathways caused by EA and the noxious stimulation.

C-Fos and c-Jun induced by EA alone provide a framework to explore the mechanism underlying EAA. Several studies tried to establish a link between EAA and c-Fos/c-Jun expression in the brain. Guo et al. [51] compared differential expression levels of c-Fos at 2 h after EA stimulation at ST36 and SP5 in rats and found that the c-Fos levels induced by a low frequency (2 Hz) were higher in ARC, PVH, periventricular nucleus of the hypothalamus, dorsomedial nucleus of the hypothalamus, VMH, supramammillary nucleus, area of the tuber cinereum, and NTS but lower in the habenular nucleus (HB), paraventricular thalamic nucleus, and GI compared to those induced by a high frequency (100 Hz). This showed that distinct neurochemical mechanisms may be involved in mediating analgesia induced by electroacupuncture of different frequencies. In Guo et al.'s study, 2 Hz EA did not induce c-Fos expression in HB, paraventricular thalamic nucleus, lateral PBN, Gi, and paragigantocellular nucleus and induced low c-Fos expression in NRM. However, Lee and Beitz [52] reported that increased c-Fos expression was induced in HB and the lateral PBN immediately after a low frequency (4 Hz) EA at ST36 in rats. Medeiros et al. [45] mapped neuronal activity with c-Fos immunohistochemistry in animals unanesthetized but habituated to repeated immobilization for 6 days and found that c-Fos expression was upregulated in HB and GI of rats at 1 h after 2 Hz EA at ST36. These results were inconsistent with those reported by Guo et al. [51]. This discrepancy may be caused by the different sampling time or habituation status of the animals.

Normally c-Fos and c-Jun are synchronously expressed after peripheral stimulations. However, Wang et al. [10] used 98 Hz EA at a set of ST36-GB39 and ST32-SP6 in cats and found increased expression of c-Jun but the absence of c-Fos in the specific spinal segments at 7 and 14 days after EA. Cullinan et al. [53] compared the patterns of c-Fos and c-Jun expression in the rat brain at 30 min following acute stress and found that c-Jun expression was significantly higher in some nuclei or areas such as the basolateral amygdala and PVH, compared with c-Fos expression. These results show that c-Fos expression is inconsistent with c-Jun expression in some areas of the CNS or under some specific circumstance. The time course of c-Fos and c-Jun accumulation and decay may be different in various brain regions. In addition to the formation of heterodimers with c-Fos, C-Jun can form homodimers, which serve as transcription factors by binding to the activator protein-1 site in target genes [54, 55]. Therefore, the detection of c-Jun expression might compensate for the deficiency of c-Fos. Both c-Fos and c-Jun were used for mapping neural activity and for understanding the mechanisms underlying EAA in this study.

In the present study, c-Fos and c-Jun were expressed to some extent in some nuclei in the control group, which is similar to the findings of previous reports, except that of Guo et al. [51]. EA increased c-Fos or c-Jun expression in most analgesia-related nuclei or areas, which is also consistent with previous studies [52, 56, 57]. The levels at which c-Fos-IR and c-Jun-IR neurons were induced by EA in the brain are frequency-dependent. Compared with 100 Hz EA, 2 Hz EA induced more c-Fos-IR neurons in PVH, VMH, ARC, and AB; more c-Jun-IR neurons in PVH and AB; fewer c-Fos-IR neurons in HL and SNC; and fewer c-Jun-IR neurons in ACB, ASL, and CAU. The c-Fos expression induced by low and high frequencies in VMH, ARC, HL, or SNC in our experiment is consistent with that reported by Guo et al. [51] and Lee and Beitz [52]. EA induced c-Jun-IR neurons to increase in ACB, ASL, CAU, PVH, and AB, demonstrating that the detection of c-Jun-IR expanded the profile of EA-activated nuclei. Sixty Hz EA increased more c-Fos-IR or c-Jun-IR neurons than 2 Hz or 100 Hz EA in most of the measured nuclei or areas. These results indicate that 60 Hz EA activated more extensive neural circuits in goats, which may be a better explanation for its analgesic phenotype (pain threshold).

Neuronal axons distribute signals beyond a given structure and are involved in communication between different brain structures or may be involved in the transmission of signals to far away brain structures or to the spinal cord. The positive signal of c-Fos or c-Jun can only reflect the activities of the neuronal cell bodies rather than the neuropil. In addition, EA may produce some inhibitory effects on neuronal activities that could not be detected by the Fos and Jun methods. However, the increased expression of c-Fos and c-Jun provides a potent means for mapping activated neuronal circuitries and understanding the neural mechanisms underlying EAA in our study.

## 5. Conclusion

Compared with 100 Hz EA, 2 Hz EA induced more c-Fos-IR neurons in PVH, VMH, ARC, and AB; more c-Jun-IR neurons in PVH and AB; fewer c-Fos-IR neurons in HL and SNC; and fewer c-Jun-IR neurons in ACB, ASL, and CAU. Sixty Hz EA increased the pain threshold and induced more c-Fos-IR or c-Jun-IR neurons in most of the measured nuclei or areas than 2 Hz or 100 Hz EA. It was suggested that 60 Hz EA activated more extensive neural circuits in goats, which may contribute to optimal analgesic effects and species variation.

## Figures and Tables

**Table 1:** C-Fos and c-Jun immunoactivities induced by different frequencies in the brain (mean ± SD, *n* = 7).

Nuclei and areas	c-Fos/c-Jun	Blank control	0 Hz/sham control	2 Hz	60 Hz	100 Hz
ACB	c-Fos	13.30 ± 1.49^b^	13.91 ± 2.36^b^	17.04 ± 1.28^b^	31.96 ± 4.91^a^	14.81 ± 2.49^b^
	c-Jun	14.62 ± 2.22^c^	15.26 ± 1.99^c^	29.21 ± 5.08^b^	39.90 ± 7.45^a^	43.37 ± 2.21^a^

ASL	c-Fos	21.64 ± 2.65^b^	20.15 ± 2.67^b^	25.55 ± 3.45^b^	48.88 ± 5.32^a^	19.56 ± 1.41^b^
	c-Jun	33.32 ± 3.61^b^	33.41 ± 3.67^b^	41.35 ± 8.51^b^	83.86 ± 4.10^a^	83.81 ± 3.83^a^

CAU	c-Fos	15.71 ± 2.21^bc^	13.06 ± 1.48^c^	14.40 ± 2.49^bc^	49.09 ± 5.62^a^	19.56 ± 3.01^b^
	c-Jun	10.06 ± 1.60^b^	9.73 ± 1.33^b^	9.56 ± 2.05^b^	25.36 ± 2.27^a^	25.39 ± 2.46^a^

SON	c-Fos	27.43 ± 4.54^b^	21.90 ± 4.82^b^	23.36 ± 3.01^b^	71.01 ± 11.30^a^	31.81 ± 2.99^b^
	c-Jun	28.81 ± 3.75^c^	28.57 ± 2.70^c^	37.00 ± 9.42^bc^	96.79 ± 5.36^a^	44.36 ± 5.87^b^

PVH	c-Fos	23.29 ± 7.79^c^	22.21 ± 3.24^c^	49.45 ± 7.31^b^	67.49 ± 10.03^a^	30.63 ± 11.70^c^
	c-Jun	25.88 ± 2.79^d^	26.87 ± 3.04^d^	52.09 ± 4.62^b^	67.44 ± 4.61^a^	39.97 ± 4.13^c^

VMH	c-Fos	29.32 ± 2.36^c^	29.16 ± 3.11^c^	48.43 ± 4.85^b^	61.15 ± 2.49^a^	35.12 ± 5.28^c^
	c-Jun	21.91 ± 3.99^c^	20.22 ± 3.21^c^	48.08 ± 7.88^b^	69.22 ± 11.22^a^	48.24 ± 7.13^b^

ARC	c-Fos	29.88 ± 3.22^c^	30.41 ± 3.50^c^	61.22 ± 2.98^b^	125.01 ± 21.55^a^	43.71 ± 3.66^c^
	c-Jun	24.08 ± 2.72^c^	24.32 ± 2.30^c^	50.39 ± 7.29^bc^	90.91 ± 13.14^a^	60.96 ± 10.14^b^

AB	c-Fos	18.08 ± 5.14^b^	17.31 ± 4.47^b^	72.34 ± 7.81^a^	70.51 ± 6.40^a^	27.76 ± 9.53^b^
	c-Jun	13.06 ± 2.07^c^	14.31 ± 1.74^c^	55.38 ± 5.93^a^	38.36 ± 5.01^b^	21.63 ± 9.09^c^

HL	c-Fos	11.81 ± 0.83^d^	11.88 ± 1.16^d^	29.59 ± 4.88^c^	78.66 ± 5.08^a^	50.88 ± 2.37^b^
	c-Jun	40.30 ± 4.15^a^	40.94 ± 4.63^a^	46.89 ± 3.92^a^	50.99 ± 11.76^a^	43.35 ± 5.79^a^

PAG	c-Fos	20.73 ± 2.87^c^	20.29 ± 2.89^c^	47.13 ± 6.98^b^	79.43 ± 10.80^a^	44.89 ± 9.14^b^
	c-Jun	26.45 ± 2.68^c^	27.49 ± 2.59^c^	44.58 ± 4.02^b^	70.58 ± 9.01^a^	36.76 ± 4.50^b^

SNC	c-Fos	25.77 ± 4.13^c^	24.75 ± 3.87^c^	33.51 ± 9.79^c^	83.25 ± 14.48^a^	49.97 ± 10.01^b^
	c-Jun	34.10 ± 3.88^b^	32.15 ± 2.88^b^	39.91 ± 6.93^b^	51.85 ± 7.31^a^	38.96 ± 7.84^b^

PBN	c-Fos	31.60 ± 4.22^c^	30.14 ± 4.79^c^	63.75 ± 12.03^b^	77.43 ± 9.18^a^	56.59 ± 4.11^b^
	c-Jun	29.38 ± 3.56^c^	27.31 ± 4.67^c^	40.75 ± 11.78^bc^	75.13 ± 10.78^a^	56.95 ± 5.23^b^

LC	c-Fos	25.36 ± 3.62^c^	25.95 ± 2.96^c^	63.60 ± 10.51^b^	103.49 ± 5.93^a^	53.81 ± 5.32^b^
	c-Jun	31.23 ± 2.95^b^	30.80 ± 3.41^b^	61.75 ± 6.62^a^	67.67 ± 10.10^a^	68.63 ± 6.15^a^

NRM	c-Fos	7.79 ± 1.73^c^	7.18 ± 1.66^c^	16.03 ± 2.71^b^	25.39 ± 3.64^a^	18.62 ± 3.51^b^
	c-Jun	26.19 ± 2.26^b^	26.64 ± 3.20^b^	31.37 ± 3.71^b^	45.74 ± 6.82^a^	33.62 ± 7.72^b^

NTS	c-Fos	27.76 ± 3.08^c^	22.63 ± 4.15^c^	34.94 ± 2.49^b^	51.27 ± 4.53^a^	38.90 ± 3.96^b^
	c-Jun	30.45 ± 3.56^c^	29.29 ± 2.59^c^	56.40 ± 5.30^b^	75.18 ± 4.61^a^	60.04 ± 5.09^b^

GI	c-Fos	15.70 ± 2.71^c^	16.03 ± 2.30^c^	36.65 ± 4.66^b^	56.81 ± 9.74^a^	45.42 ± 6.28^b^
	c-Jun	26.76 ± 2.37^c^	26.67 ± 3.73^c^	34.10 ± 9.60^bc^	52.54 ± 5.26^a^	42.49 ± 3.43^b^

The blank control refers to the group in which the goats were restrained in the same way as the EA-treated goats but without needling and electric stimulation. Values with different letters within the same row differ significantly (*P* < 0.05).
